# Insomnia

**Published:** 1889-10

**Authors:** 


### INSOMNIA.

Are you afflicted with insomnia ? Perhaps you have too much time for sleep. Perhaps you depend too much on sleep for rest and recuperation. For sleep is not the sole rest of used-up nerves. Sociability, congeniality, and the enjoyment of good company rest the body quite as much as sleep. The dreary monotony of life in many a household, involving this tumbling into bed with the mechanical regularity of a machine at nine or ten o'clock in the evening, does not always rest weary bodies " Early to bed and early to rise " does not always make a man healthy, wealthy, or wise. Numbers of organizations are only capable of five or six hours' sleep at a time, and their early lying down to rest is often succeeded by an early waking up and a consequent restless tossing for hours proceeding daybreak. The practicers of punctuality are often surprised, after breaking their own cast-iron rules, and passing two or three later hours of mirth and jollity past their usual bed-time, to find themselves even more refreshed in the morning than usual. The relaxation or sociability has rested them more than would sleep or an attempt to sleep But these are conditions not so easily reached in the average family. In fashionable life we have a formal, exhausting, and mechanical evening, of more or less dissipation. On the other hand, the evenings of great numbers of families are monotonously humdrum. They involve the assemblage of the same people, the same surroundings, the same paterfamilias yawning over his paper, and same querulous mamma overladen with family cares. Fresh people with fresh thought, fresh atmosphere, anything to stir up and agitate the pool of domestic stagnation, are sadly needed and sadly scarce. There needs to be, also, a constant succession of such fresh people to bring about these results. The world is full of men and women, and in a better regulated life it would be the business after the day's work was done to entertain each other, and give each other fresh life. As it is now, hundreds if not thousands of our households are little beter than cells for the incarceration of each family. Thousands are thus worn out prematurely from utter lack of domestic recreation. There might be written over the graves of hundreds of thousands, "Bored to death by the stagnation of domestic life."—*The Christian at Work.*

